# Supplementary material for: Development of a Simple DNA Extraction Method and Candida Pan Loop-Mediated Isothermal Amplification Assay for Diagnosis of Candidemia
Source: Pathogens. 2022 Jan 18;11(2):111. doi: 10.3390/pathogens11020111 (PMC8878442; doi:10.3390/pathogens11020111)
Supplement: Supplementary file 1 [file pathogens-11-00111-s001.zip › pathogens-1558963-supplementary.pdf]

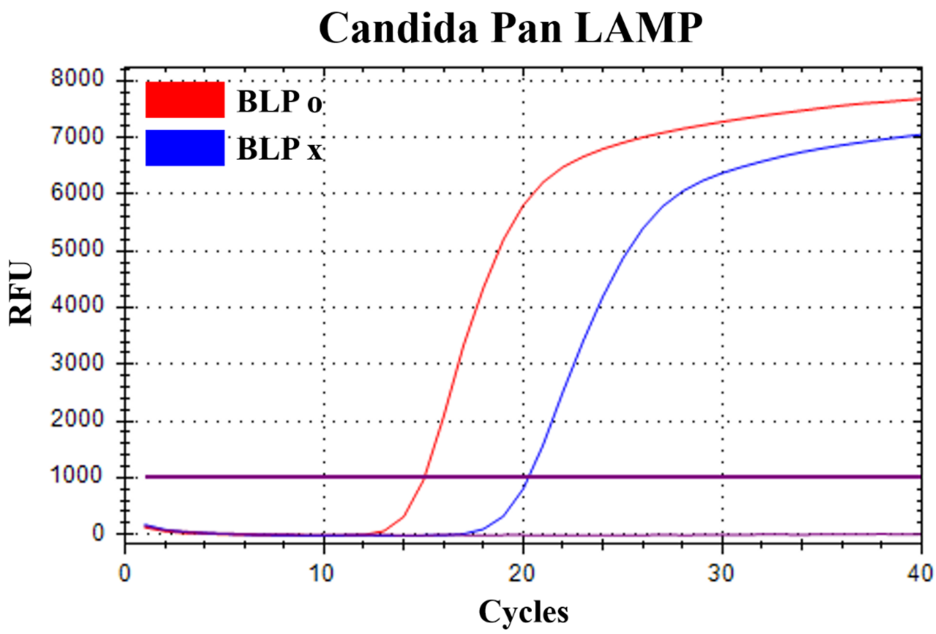

Figure S1. Comparison of ct values of Candida Pan LAMP assay with/without BLP.

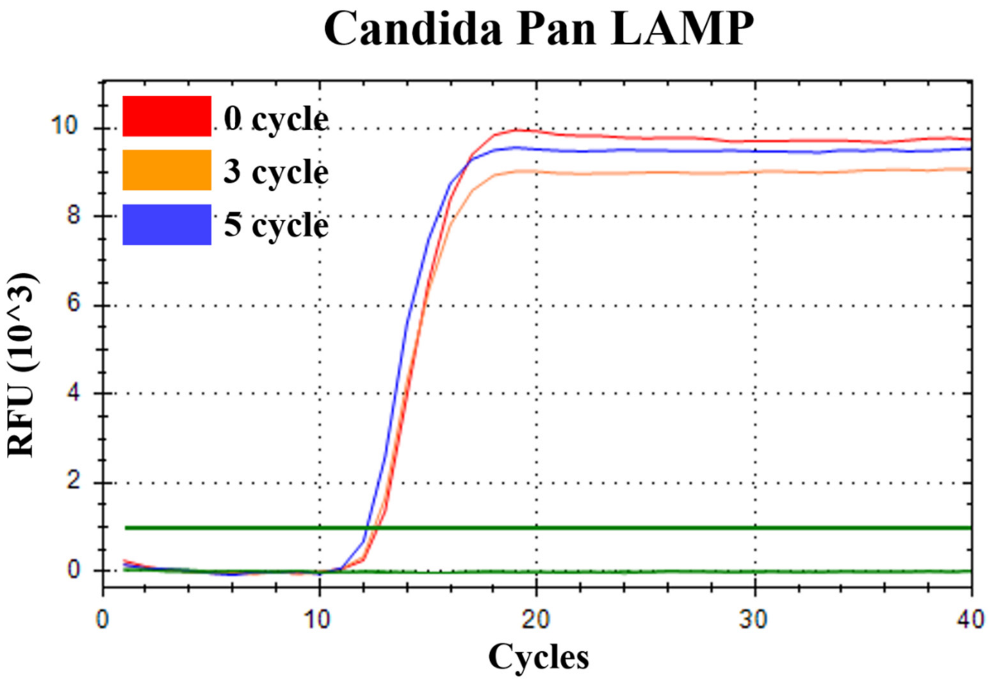

Figure S2. Comparison of ct values of lamp assay according to bead beat cycles (0, 5 and 10 cycles) in Chelex-100/boiling DNA extraction.
